# Peroxisome proliferator-activated receptor gamma ligand-mediated apoptosis of hepatocellular carcinoma cells depends upon modulation of PI3Kinase pathway independent of Akt

**DOI:** 10.1186/1750-2187-5-20

**Published:** 2010-12-13

**Authors:** Prajna Mishra, Suresh K Paramasivam, Ramesh P Thylur, Ajay Rana, Basabi Rana

**Affiliations:** 1 Department of Medicine, Division of Gastroenterology, Hepatology and Nutrition, Loyola University, Chicago, 2160 South First Avenue, Maywood, IL 60153, USA; 2 Department of Molecular Pharmacology & Therapeutics, Loyola University Chicago, 2160 South First Avenue, Maywood, IL 60153, USA; 3 Hines VA Medical Center, Hines, IL, USA

## Abstract

**Background:**

Ligands of Peroxisome proliferator-activated receptor gamma (PPARγ) can inhibit growth and promote apoptosis in various cancer cells, and thus have the potential to be utilized as anticancer drugs. This potential however, has been seriously challenged by observations that they can lead to tumor promotion in some cancer models, possibly due to activation of different signaling mechanisms in various tumor environments. Elucidation of the specific signaling events that modulate PPARγ ligand-mediated events is thus critical to increase their efficacy. The studies described here were designed to elucidate the signaling pathway(s) that modulate the apoptotic potential of Troglitazone (TRG), an artificial PPARγ ligand in hepatocellular carcinoma (HCC) cells.

**Results:**

Our results indicate that the apoptotic potential of TRG was regulated by the presence or absence of serum in the media. When added in serum-containing media, TRG inhibited proliferation and cyclin D1 expression, but was unable to induce any apoptosis. However, TRG's apoptotic potential was induced significantly when added in serum deficient media, as indicated by increased PARP and Caspase-3 cleavage and results from apoptosis assay. Furthermore, TRG-induced apoptosis in serum deficient media was associated with a dramatic reduction in PI3Kinase downstream target Akt^Ser473 ^and FoxO1^Thr24^/FoxO3a^Thr32 ^phosphorylation. On the contrary, there was an increase of PI3K-induced Akt^Ser473 ^and FoxO1^Thr24^/FoxO3a^Thr32 ^phosphorylation involving Pak, when TRG was added in serum-containing media. Pharmacological inhibition of PI3Kinase pathway with LY294002 inhibited Akt^ser473 ^phosphorylation and sensitized cells towards apoptosis in the presence of serum, indicating the involvement of PI3K in apoptosis resistance. Interestingly, pharmacological inhibition or siRNA-mediated knockdown of Akt or inhibition of Pak was unable to sensitize cells towards TRG-induced apoptosis in the presence of serum. Similarly, TRG was unable to induce apoptosis in the Akt1-KO, Akt1&2-KO MEFs in serum-containing media.

**Conclusion:**

These studies indicate that TRG-induced apoptosis is modulated by PI3K pathway in a novel Akt-independent manner, which might contribute to its tumor promoting effects. Since PI3K activation is linked with various cancers, combination therapy utilizing TRG and PI3K inhibitors has the potential to not only increase the efficacy of TRG as a chemotherapeutic agent but also reduce its off target effects.

## Background

Hepatocellular carcinoma (HCC) is one of the most common forms of gastrointestinal (GI) cancers, and thus a major cause of death, worldwide [1]. Neoplastic hepatic cells not only loose their ability to regulate growth, but they also become dedifferentiated and thereby loose their differentiated function. The average survival time of patients with advanced nonresectable form of the disease is very small [2], and thus development of safer noninvasive therapeutic approaches is critical to combat this deadly disease.

Peroxisome proliferator-activated receptors (PPARs) are ligand-activated transcription factors, involved in regulating many important biological processes, including growth, differentiation, apoptosis [3]. The PPAR family comprises of three distinct members PPARα, PPARδ, PPARγ, which function via forming heterodimers with retinoid X receptor (RXR). PPARγ has been studied extensively and it is now well established that this molecule plays a prominent role in regulating differentiation of adipocytes and macrophage foam cells [4,5]. Ligands of PPARγ include naturally occurring compounds such as fatty acids and prostaglandin D2 metabolite 15-deoxy-Δ12,14-prostaglandin J2 (15d-PGJ2)[6], as well as the artificial ones known as Thiazolidinediones. These Thiazolidinediones include Troglitazone (TRG), Ciglitazone, Pioglitazone, which are also known to improve insulin sensitivity [7,8], some of which are currently used for treating type II diabetes [9].

More recent studies indicate a new and emerging role of PPARγ in regulating growth of cancer cells [9]. Functionally active PPARγ is expressed in a variety of cancer cells, including those from liposarcomas, colon, breast, prostate and liver, which respond to Thiazolidinedione treatment via inducing growth arrest [10-13], However, studies with *in vivo *cancer models have provided conflicting results, thus questioning the efficacy of PPARγ ligands as chemotherapeutic agents and raising concerns regarding the long-term term use of these as diabetic drugs. Agonist-induced activation of PPARγ in a colon cancer xenograft model showed reduction of tumor growth [14], whereas it resulted in tumor promotion when PPARγ was activated in a genetic model of colon cancer (APC^Min ^mice) [15,16]. In the intestinal epithelial cells, PPARγ was shown to induce EMT [17], a process that is known to mediate cancer cell migration, invasion as well as acquisition of stem cell properties [18]. In a separate study, transgenic mice overexpressing a constitutive active form of PPARγ was shown to exacerbate mammary tumor development [19]. Treatment of mice lacking one copy of the PPARγ gene with the carcinogen azoxymethane showed a significant increase in the frequency of colon tumors [20], while other studies with mice having a breast epithelium specific ablation of PPARγ showed no increase in breast tumors [21]. TRG was also shown to be effective in reducing tumor growth in mouse HCC cell xenografts [22], and inducing differentiation in patients with advanced liposarcomas [23]. The reasons behind these paradoxical effects are still unknown and need to be elucidated as it suggests that PPARγ-mediated pathways are likely modulated by specific downstream signaling events in various tumor environments.

The process of apoptosis is tightly controlled by complex signaling networks that involve activation and inhibition of specific downstream target proteins. Majority of the cancer cells acquire characteristics to alter these regulatory signaling networks, leading to evasion of apoptosis and promotion of survival. Therapeutic approaches that can override these alterations and produce cancer cell apoptosis have the potential to be developed as effective drugs for cancer treatment. One such signaling pathway is the Phosphatidylinositol-3 Kinase (PI3K)/Akt pathway, which is frequently activated in cancer [24-26] and is linked with cancer cell survival [26,27]. The effect of PPARγ agonists on cellular apoptosis is also variable, with increased apoptosis in some cancer cells [28-30] and none in others [31], which might be due to modulation of the signaling molecules by PPARγ ligands in various cancer pathways.

In an effort to better understand the effects of PPARγ on HCC cell apoptosis, we focused on elucidating the signaling pathway(s) that modulate the apoptotic potential of TRG, an artificial PPARγ ligand. Our results indicate that TRG (when added in serum-containing media) can induce growth arrest associated with a reduction of cyclin D1, PCNA (proliferating cell nuclear antigen) as well as p21^CIP1 ^and p27^KIP1 ^expression. However, TRG was unable to induce any apoptosis in these cells when added in serum-containing media, which was associated with an increase in Akt^Ser473 ^and FoxO1^Thr24^/FoxO3a^Thr32 ^phosphorylation, indicating activation of PI3K/Akt axis. This increase in Akt^Ser473 ^phosphorylation seems to involve Pak, since pretreatment with a Pak inhibitor abolishes TRG-induced phosphorylation of Akt^Ser473^. Treatment with TRG in serum-deficient media induced potent apoptosis as evident from an increase in Caspase-3 and PARP cleavage and the results from apoptosis assays. Elucidation of the upstream signaling pathways indicated that TRG-mediated apoptosis in serum-deficient media is associated with a dramatic reduction in Akt^Ser473 ^and FoxO1^Thr24^/FoxO3a^Thr32 ^phosphorylation. Pharmacological inhibition of PI3Kinase pathway inhibited TRG-induced increase of Akt^Ser473 ^phosphorylation and sensitized cells to apoptosis even in the presence of serum. However, pharmacological inhibition or siRNA-mediated knockdown of Akt was unable to sensitize cells to TRG-induced apoptosis in the presence of serum. Similarly, TRG was unable to induce apoptosis in the MEFs with either Akt1 or Akt1/2 knockout, suggesting that TRG-mediated apoptosis is modulated by PI3K pathway in an Akt-independent manner. In addition, knockdown of PPARγ expression although unable to sensitize the cells to TRG-induced apoptosis in serum-containing media, partially reduced TRG-induced increase of Akt^Ser473 ^phosphorylation suggesting the latter to be PPARγ-dependent effect of TRG.

## Results

### Effect of TRG on HCC cell proliferation

**Figure 1:**
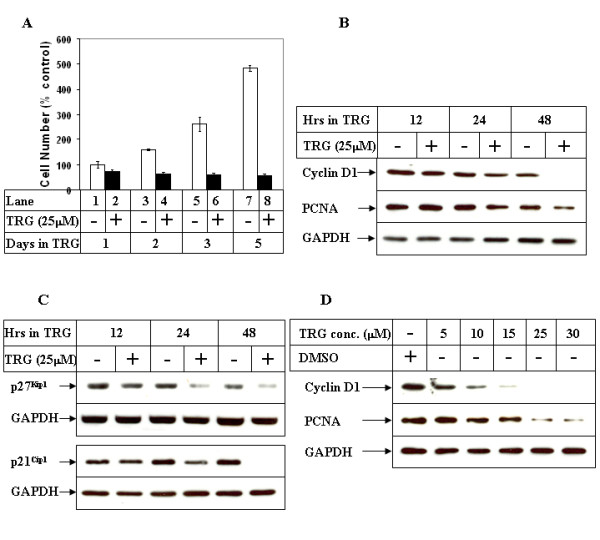
**Effect of TRG on HCC cell proliferation**. ***(A) ***Subconfluent Huh-7 cells were plated on 6 well plates in regular growth medium. Next day, they were treated with either DMSO (-) or 25 μM TRG (+) and harvested at the indicated time intervals. The cell numbers were determined and represented as % control considering the DMSO-treated sample of 24 hours as 100%. Cells were plated in triplicate for each time point and each experiment was repeated at least twice. ***(B) ***Cells were treated as in A for the indicated time periods, following which they were harvested and total protein was extracted. Western Blot analysis of the cell extracts was then performed with antibodies against Cyclin D1, PCNA and GAPDH (as control). ***(C) ***The cells were treated as in B and cell extracts analyzed by Western Blots with the indicated antibodies. ***(D) ***Huh-7 cells were treated with either DMSO or increasing concentration of TRG for 48 hours, followed by Western Blot analysis with the indicated antibodies.

Our earlier results showed that TRG-mediated activation of PPARγ can induce growth arrest at G_1_/S stage [32]. Similarly, studies with Huh-7 HCC cells showed a TRG-mediated inhibition of cell proliferation with time (Figure 1A). Western Blot analysis carried out with these cells showed a TRG-induced decrease in the expression of cyclin D1 and PCNA in a time (Figure 1B) and dose dependent manner (Figure 1D). Surprisingly, the expression of the cyclin dependent kinase inhibitors (CDKIs) p21^CIP1 ^and p27^Kip1 ^(known to mediate growth arrest), also showed a TRG-dependent decrease (Figure 1C), coinciding with the time of growth arrest. These results indicated that TRG was capable of inhibiting proliferation of HCC cells, which is associated with a reduced expression of cyclin D1, PCNA as well as p21^CIP1 ^and p27^Kip1^.

### Effect of PI3Kinase Pathway on TRG-induced growth arrest of HCC cells

**Figure 2:**
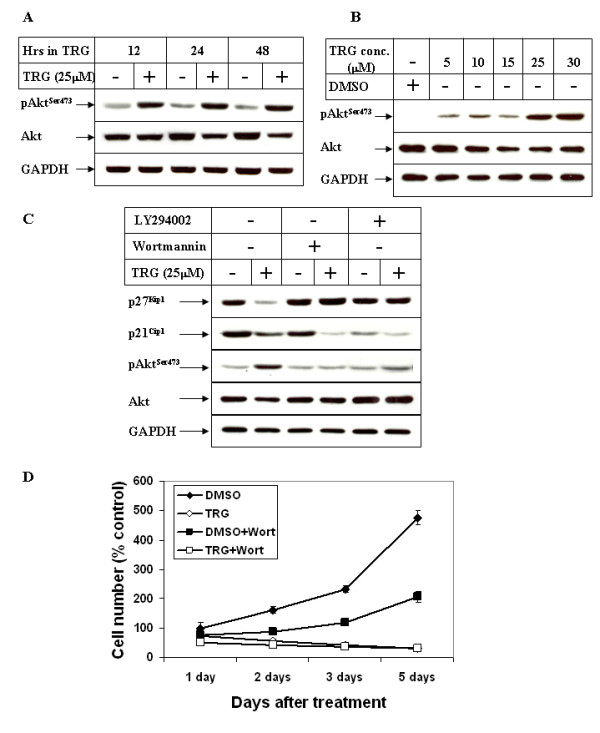
**Role of PI3Kinase pathway on TRG-induced cell growth arrest**. ***(A) ***Subconfluent Huh-7 cells were treated as in 1B or ***(B) ***as in 1D, followed by Western Blot analysis of the cell extracts with antibodies against pAkt^Ser473^, Akt and GAPDH. ***(C) ***Huh-7 cells were treated in the absence (-) or presence (+) of 25 μM TRG for 24 hours following a 1 hour pretreatment with either none (lanes 1 & 2), or 1 μM Wortmannin (lanes 3 & 4) or 5 μM LY294002 (lanes 5 & 6). Western Blot analysis was then performed with the antibodies indicated. ***(D) ***Subconfluent Huh-7 cells were treated with DMSO, 25 μM TRG, DMSO + 1 μM Wortmannin, or TRG + 1 μM Wortmannin for the indicated time intervals, following a 1 hour pretreatment with Wortmannin. Cell proliferation assay was performed as described in 1A.

Several earlier reports suggested that phosphatidylinositol-3 Kinase (PI3K)/Akt pathway is involved in down-regulating p27^Kip1 ^expression [33,34] and regulating p21^CIP1 ^localization [35], raising the possibility that TRG might regulate these proteins via modulating the PI3K/Akt pathway. Western Blot analysis performed with TRG-treated cell extracts (spanning the period of growth arrest) showed an increase in Akt^Ser473 ^phosphorylation following stimulation with TRG in a time and dose dependent manner (Figures 2A &2B respectively). Since Akt^Ser473 ^phosphorylation is required for full Akt activation downstream of PI3K pathway, this indicated an activation of PI3K/Akt pathway following treatment with TRG. In order to determine whether the growth arrest induced by TRG involved PI3K/Akt pathway, studies were designed next following pretreatment with two different pharmacological inhibitors of PI3K, Wortmannin and LY294002. Pretreatment with PI3K inhibitors attenuated TRG-mediated induction of Akt^Ser473 ^phosphorylation, indicating the involvement of PI3K in inducing Akt^Ser473 ^phosphorylation following TRG addition (Figure 2C, pAkt^Ser473 ^panel, compare lanes 4 & 6 with 2). In addition, PI3K inhibitors also antagonized down-regulation of p27^Kip1 ^expression but not p21^CIP1 ^(Figure 2C, p27^Kip1 ^and p21^CIP1 ^panels), suggesting the involvement of this signaling pathway in TRG-induced down-regulation of p27^Kip1 ^expression. However, PI3K inhibition was unable to antagonize TRG-induced cell growth arrest as shown in Figure 2D. These results indicated that stimulation by TRG leads to an activation of PI3K/Akt pathway, which in turn down-regulated the expression of p27^Kip1 ^in a cell proliferation-independent manner.

### TRG-induced apoptosis in HCC cells depends upon the availability of serum

**Figure 3:**
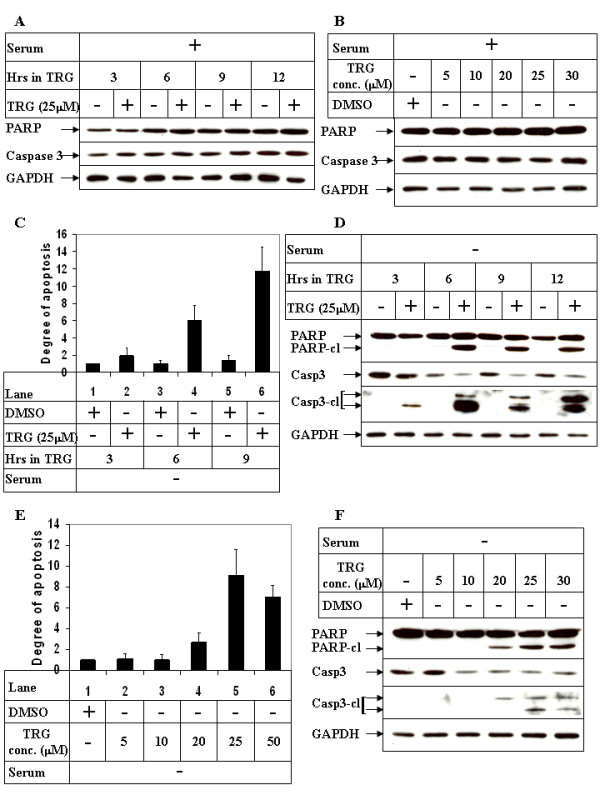
**Effect of TRG on HCC cell apoptosis in the presence or absence of serum**. ***(A) ***Subconfluent Huh-7 cells were treated with 25 μM TRG in serum-containing media for the indicated time intervals. Western Blot analysis was performed with antibodies against PARP, Caspase-3 and GAPDH (as control). ***(B) ***Huh-7 cells were treated with increasing concentrations of TRG in serum-containing media for 9 hours and subjected to Western Blot analysis as in A. ***(C) ***Huh-7 cells were treated with 25 μM TRG in serum deficient media for the indicated time intervals. At the end of incubation cells were harvested and apoptosis assays were performed using cell death detection ELISA^PLUS ^kit. The data in each set represents the mean ± S.D. of 4 independent experiments. ***(D) ***Western Blot analysis of cell extracts treated with TRG in serum deficient media for the indicated periods of time and with antibodies against PARP, Caspase-3, cleaved Caspase-3 (detects only the cleaved form) and GAPDH. ***(E) ***&***(F) ***Huh-7 cells treated with increasing concentrations of TRG in serum-deficient media were subjected to apoptosis assays using cell death detection ELISA^PLUS ^kit **(E) **or Western Blot analysis **(F)**.

Since activation of PI3K/Akt pathway has been shown to inhibit apoptosis and promote survival in many cancer cells [26], it is likely that the apoptotic potential of TRG is regulated by PI3K/Akt pathway. Interestingly, TRG when added in serum-containing media was unable to induce any apoptosis, despite being able to successfully induce cell growth arrest (Figure 1A). This is evident from the absence of PARP or Caspase-3 cleavage even with the highest concentration of TRG used (Figures 3A and 3B). This suggested that TRG-mediated cell growth arrest and apoptosis induction might be distinct from each other involving different signaling mechanisms. However, addition of TRG in serum deficient media resulted in potent apoptosis within a short time as estimated by apoptosis assays (Figures 3C) and Western Blot analysis (Figure 3D, compare PARP and Caspase-3 cleavage in - and + TRG lanes). Prominent apoptotic morphology was evident within hours of TRG treatment and resulted in almost complete cell death by 12 hours (data not shown). This apoptotic effect was maximal with 25 μM TRG as shown in Figures 3E and 3F. No apoptosis, however, was visible when cultured in serum-deficient media in the absence of TRG (Figures 3C &3D, -TRG lanes), indicating that these are TRG specific effects. Results from these studies indicate that the presence of serum (or factors in the serum) antagonize the apoptotic potential of TRG, which is reversed when TRG treatment is performed in the absence of serum. Since TRG treatment in serum-containing media resulted in an increase in Akt^Ser473 ^phosphorylation (Figures 2A &2B), via PI3K activation (Figure 2C), it was conceivable that activation of PI3K/Akt pathway antagonized TRG-induced apoptosis in the presence of serum.

### TRG treatment inhibits PI3Kinase/Akt Pathway in the absence of serum

**Figure 4:**
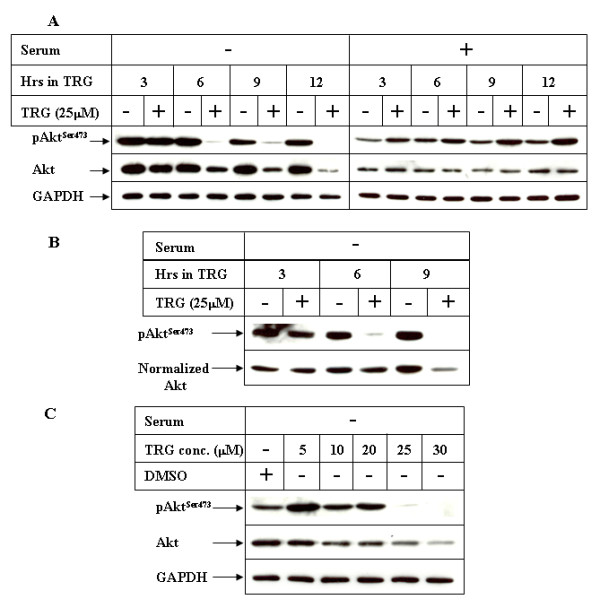
**Effect of TRG on PI3K/Akt Pathway in the presence or absence of serum**. ***(A) ***Huh-7 cells were treated with 25 μM TRG in the absence (-) or presence (+) of serum for the indicated periods of time. Equal amounts of cell extracts were analyzed by Western Blot analysis utilizing antibodies against pAkt^Ser473^, Akt or GAPDH. ***(B) ***Western Blot analysis of the cell extracts treated with TRG in serum-deficient media following normalization of total AKT levels. ***(C) ***Cells treated with increasing concentrations of TRG for 6 hours in serum deficient media were subjected to Western Blot analysis as in A.

To determine any correlation of PI3K/Akt pathway with TRG-mediated apoptosis, we first determined the status of PI3K pathway following TRG stimulation under serum deprived conditions. Western Blot analysis showed a time (Figure 4A) and dose (Figure 4C) dependent decrease in Akt^Ser473 ^phosphorylation following TRG treatment under serum deprived conditions. This is in sharp contrast to TRG-mediated increase in Akt^Ser473 ^phosphorylation in the presence of serum as shown in early time course (Figure 4A, + serum panel) and longer time course studies (Figures 2A &2B). Decrease in Akt^Ser473 ^phosphorylation in the absence of serum indicated an inhibition of PI3K/Akt pathway, which coincided with TRG-induced apoptosis (Figure 3D). Surprisingly, TRG treatment in the absence of serum also resulted in a significant decrease in total Akt expression. In order to rule out the possibility that the decrease in Akt^Ser473 ^phosphorylation was due to a corresponding decrease of total Akt expression, Western Blot analysis was performed with TRG-treated samples following normalization of total Akt levels. These results showed that Akt^Ser473 ^phosphorylation was reduced independent of total Akt expression (Figure 4B, 6 hr -/+TRG lanes, compare phospho- and total Akt panels).

**Figure 5:**
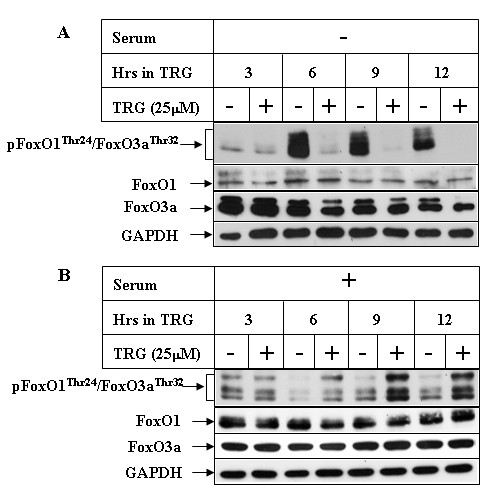
**Effect of TRG on FoxO1/FoxO3a phosphorylation in the presence or absence of serum**. Huh-7 cells were treated with 25 μM TRG for the indicated time intervals in serum deficient ***(A) ***or serum-containing ***(B) ***media. Western Blot analyses were performed with antibodies against phospho-FoxO1^Thr24^/FoxO3a^Thr32^, FoxO1, FoxO3a and GAPDH.

Since Akt activation is known to mediate cell survival via phosphorylation and inactivation of downstream proteins (FoxO1/FoxO3a), we estimated the phosphorylation status of FoxO1/FoxO3a proteins following treatment with TRG in both serum-containing and serum deprived media. Western Blot analysis was performed with an antibody against phospho-FoxO1^Thr24^/FoxO3a^Thr32 ^which detects FoxO1 when phosphorylated at Threonine 24 and FoxO3a when phosphorylated at Threonine 32, both of which are Akt phosphorylation sites [36]. The results indicated a decrease in the levels of phospho-FoxO1^Thr24^/FoxO3a^Thr32 ^following stimulation by TRG in serum deficient media (Figure 5A), which also correlated with inhibition of Akt under these conditions (Figure 4A). Similarly, addition of TRG in serum-containing media resulted in an increase in phospho-FoxO1^Thr24^/FoxO3a^Thr32 ^levels (Figure 5B) and correlated with increased Akt activation (Figure 4A). These suggested the possibility that TRG-mediated apoptosis depends upon modulation of the PI3K/Akt/FoxO1/3a axis, antagonism of which might increase its apoptotic potential.

### Inhibition of PI3K pathway sensitizes HCC cells to TRG-mediated apoptosis in the presence of serum

**Figure 6:**
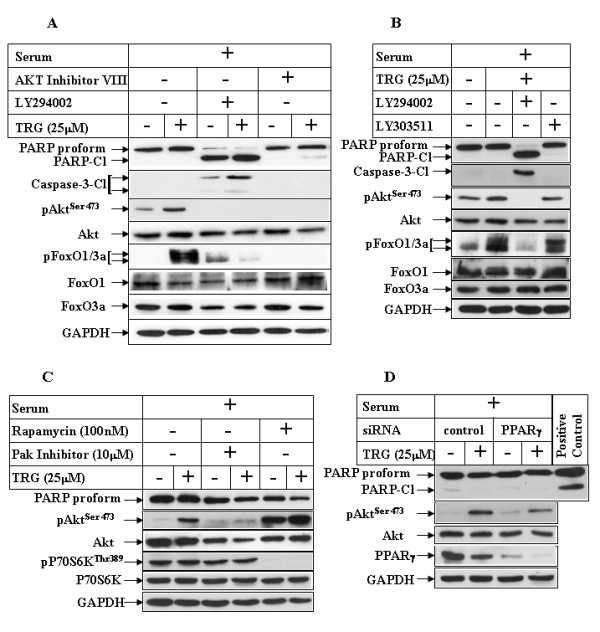
**Effect of PI3K pathway inhibition on TRG-induced apoptosis resistance in serum-containing media**. ***(A) ***Huh-7 cells were treated in the absence (-) or presence (+) of 25 μM TRG in serum-containing media for 24 hours following a 1 hour pretreatment with none (lanes 1 & 2), 50 μM LY294002 (lanes 3 & 4), or 20 μM Akt inhibitor VIII (lanes 5 & 6). Western Blot analyses were performed with the antibodies indicated. ***(B) ***Cells were treated with TRG as in A following a 1 hour pretreatment with none (lane 2), 50 μM LY294002 (lanes 3), or 20 μM LY303511 (lane 4) and analyzed by Western Blot. ***(C) ***Huh-7 cells were treated with TRG as in A, following a 1 hour pretreatment with none (lanes 1 & 2), 10 μM Pak inhibitor (lanes 3 & 4), or 100 nM Rapamycin (lanes 5 & 6). Western Blot analyses with the indicated antibodies were performed next. ***(D) ***Huh-7 cells were transfected with either a control-siRNA (lanes 1 & 2), or PPARγ-siRNA (lanes 3 & 4) for 72 hours, followed by TRG treatment in serum-containing media for an additional 24 hours. Western Blot analysis was then performed with the indicated antibodies. TRG-treated Huh-7 cell extract in serum-deficient media was used as positive control for PARP cleavage.

Studies were designed next to determine whether inhibition of PI3K pathway might sensitize cells towards TRG-induced apoptosis in the presence of serum. To address this, cells were subjected to TRG treatment in serum-containing media following a pretreatment with the pharmacological inhibitor of PI3K, LY294002. Western Blot analysis indicated an inhibition of Akt^Ser473 ^and FoxO1^Thr24^/FoxO3a^Thr32 ^phosphorylations following pretreatment with LY294002, confirming the efficacy of the inhibitor (Figure 6A). Pretreatment with LY294002 was capable of inducing apoptosis in these cells even in the presence of serum, which was increased with TRG (compare PARP and Caspase-3 cleavage in lanes 3 and 4). In order to rule out any non-specific effects of LY294002 (LY29), similar studies were also performed with LY303511 (LY30), which is a structural analog of LY29 without any inhibitory effect on PI3K pathway, and thus serves as a negative control for LY29 [37]. The results showed that TRG was capable of inducing PARP and Caspase-3 cleavage in the presence of serum only when pretreated with LY29 and not with LY30 (Figure 6B, compare lanes 3 and 4), thus confirming that the proapoptotic effects of TRG are linked with antagonism of PI3K/Akt pathway.

Several candidate kinases have been reported to phosphorylate Akt at Ser473, which include mammalian target of rapamycin complex 2 (mTORC2) [38] and p21-activated kinase-1 (Pak1) [39]. Since long-term treatment with rapamycin (drug that normally inhibits mTORC1) can also inhibit mTORC2 [40], we performed a long term (24 hour) TZD treatment in the presence of rapamycin. Rapamycin was unable to antagonize TRG-induced Akt^Ser473 ^phosphorylation and instead resulted in increased basal Akt^Ser473 ^phosphorylation (Figure 6C, compare lanes 1 & 2 with lanes 5 & 6) as also reported earlier [38], and abolished P70S6K^Thr389 ^phosphorylation (target of mTORC1). To determine whether TRG-mediated increase of Akt^Ser473 ^phosphorylation involved Pak, TRG studies were performed following pretreatment with a peptide inhibitor of Pak that disrupts PIX and Pak interaction (PAK 18) [41]. Pretreatment with Pak inhibitor abolished TRG-mediated increase of Akt^Ser473 ^phosphorylation (Figure 6C, pAkt^Ser473 ^panel, compare lanes 2 & 4). However, despite inhibiting Akt^Ser473 ^phosphorylation, Pak inhibitor was unable to induce PARP cleavage in the presence of TRG (see PARP panel). These suggested that TRG increased Akt^Ser473 ^phosphorylation via a PI3K/Pak-mediated pathway, which seem to be independent of the apoptotic pathway.

In an attempt to understand whether TRG-induced increase of Akt^Ser473 ^phosphorylation was mediated by PPARγ, small interference RNA (siRNA) studies were designed to knockdown the expression of endogenous PPARγ. Treatment with TRG showed an increase in Akt^Ser473 ^phosphorylation in the control-siRNA transfected cells (Figure 6D, compare lanes 1 & 2), which was partially reduced when PPARγ expression was knocked down (compare lanes 3 & 4). Knockdown of PPARγ expression, however, was unable to show increased apoptosis with TRG, as indicated by lack of PARP cleavage (Figure 6D, PARP panel). These suggested the involvement of PPARγ in TRG-induced phosphorylation of Akt^Ser473 ^and possibly not in the apoptosis pathway.

### PI3K antagonizes TRG-induced apoptosis independent of Akt

**Figure 7:**
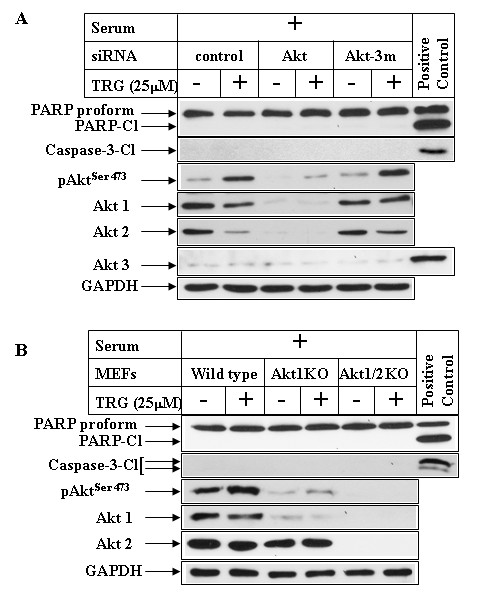
**Effect of Akt inhibition on TRG-induced apoptosis resistance in serum-containing media**. ***(A) ***Subconfluent Huh-7 cells were transfected with either control-siRNA (lanes 1 & 2), AKT-siRNA (lanes 3 & 4), or Akt-3m-siRNA (lanes 5 & 6) for 72 hours followed by treatment with 25 μM TRG for 24 hours in serum-containing media. Western Blot analysis was then performed with the antibodies indicated. ***(B) ***MEFs from Wild type (lanes 1 & 2), Akt1 KO (lanes 3 & 4) or Akt1/2 KO (lanes 5 & 6) mice were treated with 25 μM TRG in serum-containing media for 24 hours followed by Western Blot analysis. TRG-treated Huh-7 cell extracts in serum-deficient media were used as positive controls for PARP and Caspase-3 cleavage in A & B and WT-MEF extract as positive control for Akt3 in A.

To gain more insight regarding the molecules downstream of PI3Kinase pathway that might be involved in antagonizing the apoptotic potential of TRG in serum-containing media, we focused on Akt, due to its role in promoting cell survival. Surprisingly, however, apoptosis studies designed following pharmacological inhibition of Akt (with Akt Inhibitor VIII) was unable to sensitize the cancer cells to TRG-induced apoptosis in the presence of serum (Figure 6A, compare PARP and Caspase-3 cleavage, lanes 5 and 6), despite complete inhibition of the phosphorylation of Akt downstream targets FoxO1^Thr24^/FoxO3a^Thr32 ^(pFoxO1/3a panel). This indicated the possibility that PI3Kinase pathway inhibits TRG-induced apoptosis independent of Akt activation. To demonstrate conclusively that this is in fact Akt independent, experiments were performed following siRNA-induced knockdown of Akt expression. This was achieved by using an Akt-siRNA sequence that can knockdown the expression of both human Akt1 and 2 [42], which are the two major Akt isoforms expressed in these cells (Figure 7A, compare lane 1 in Akt1/Akt2/Akt3 panels). Overexpression of Akt-siRNA (Akt row) significantly reduced the expression of endogenous Akt1 and 2 (lanes 3, 4), whereas a control-siRNA (control, lanes 1, 2) or an Akt-3m-siRNA sequence containing 3 mismatches against the Akt target sequence (Akt-3m, lanes 5, 6) were unable to reduce Akt1 and 2 expression. In these studies, knockdown of Akt expression was unable to sensitize these cells to TRG-induced apoptosis in the presence of serum (Figure 7A, compare PARP and Caspase-3 cleavage in lanes 2, 4, 6). To confirm the participation of Akt, TRG studies were also performed with MEFs from Akt-WT, Akt1-KO and Akt1&2-KO animals. These showed that absence of either Akt1 or both Akt1&2 was still unable to sensitize these MEFs to TRG-induced apoptosis when added in the presence of serum (Figure 7B, compare PARP and Caspase-3 cleavage in lanes 2, 4, 6), despite a complete absence of Akt^ser473 ^phosphorylation. These studies confirmed that PI3K antagonizes TRG-induced apoptosis in an Akt-independent manner.

### Involvement of PI3K pathway in modulating TRG-induced apoptosis in other HCC cells

**Figure 8:**
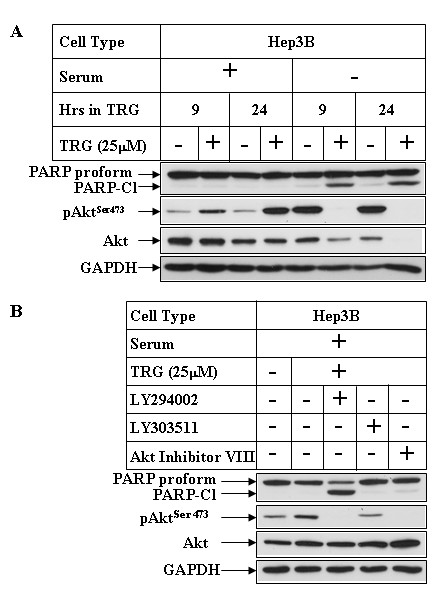
**Effect of PI3K and Akt inhibition on TRG-induced apoptosis resistance in Hep3B cells**. ***(A) ***Subconfluent Hep3B cells were treated with 25 μM TRG in the presence (+) or absence (-) of serum for the indicated periods of time. Western Blot analyses were performed with the antibodies indicated. ***(B) ***Western analysis of Hep3B extracts treated with TRG in serum-containing media for 24 hours following a 1 hour pretreatment with none (lane 2), LY294002 (lane 3), LY303511 (lane 4), or Akt inhibitor VIII (lane 5).

To determine whether PI3K modulated TRG-induced apoptosis in other HCC cells, studies were designed with Hep3B HCC cell line. As shown earlier in Huh-7 cells, treatment with TRG in the presence of serum lead to an increase in Akt^Ser473 ^phosphorylation (Figure 8A, pAkt^473 ^panel compare lanes 1 & 2 and 3 & 4) mediated via activation of PI3K pathway (Figure 8B, compare lanes 2 & 3). This is however, reversed when treated with TRG in serum deficient media resulting in a potent inhibition of Akt^Ser473 ^phosphorylation (Figure 8A, compare lanes 5 & 6 and 7 & 8). Similarly, TRG was unable to induce any PARP cleavage when added in serum-containing media (Figure 8A lanes 1-4), which was induced when added in serum deficient media (lanes 5-8). Furthermore, LY29-mediated inhibition of PI3K pathway sensitized these cells to TRG-induced apoptosis in serum-containing media (Figure 8B, PARP panel, compare lanes 2 & 3). Pretreatment with the nonspecific inhibitor LY30 (lane 4) or Akt inhibitor (lane 5) were unable to induce any PARP cleavage as was also shown earlier in Huh7 cells. These studies suggest that PI3K modulation of TRG-induced apoptosis is a generalized event in various HCC cells.

## Discussion

Studies in the recent years revealed the possibility of utilizing PPARγ ligands as cancer chemotherapeutic drugs [43]. This possibility however, has been challenged by the fact that these ligands resulted in tumor promotion in animal models of colon cancer [15,16]. In addition, overexpression of a constitutive active form of PPARγ promoted breast tumor development [19]. In terms of the cellular effects mediated by PPARγ in cancer cells, its role on growth arrest has been fairly well established, while significant controversy still exist regarding its role in mediating apoptosis. This is evident from multiple studies showing induction of cellular apoptosis by PPARγ ligands [28,30], while others [31] showing no apoptosis following Thiazolidinedione treatment. These observations indicated the possibility that specific signaling pathways operating in different tumor microenvironments might be modulating the apoptotic potential of these ligands. It is thus critical to understand the detailed signaling pathways that modulate the apoptotic potential of PPARγ ligands, targeting of which can increase their efficacy towards cancer treatment. The signaling pathway, most extensively studied in the recent years due to its close involvement in promoting cancer cell survival is the PI3K/Akt pathway [26], thus making it an important target for cancer drugs [27]. In fact, aberrant activation of PI3K/Akt pathway has been reported in multiple cancers [26,24,25]. To determine whether PI3K was involved in modulating PPARγ ligand-induced apoptosis, we designed studies with TRG, an artificial PPARγ ligand.

Our studies indicated that treatment of the HCC cells with TRG results in growth arrest associated with a reduced expression of the growth specific proteins cyclin D1 and PCNA. Surprisingly, however, TRG treatment also resulted in a decrease in the expression of CDKIs p27^Kip1 ^and p21^CIP1^, coinciding with the period of growth arrest. Activation of PI3K/Akt pathway has been shown to inhibit the expression of p27^Kip1 ^[33] and regulate the localization of p21^CIP1 ^[35]. Interestingly, TRG treatment of HCC cells in the presence of serum resulted in increased Akt^Ser473 ^phosphorylation in a time and dose dependent manner. This was also associated with increased phosphorylation of FoxO1^Thr24^/FoxO3a^Thr32 ^(downstream targets of Akt), and thus indicating an activation of PI3K/Akt axis. To understand any contribution of PI3K on TRG-induced growth arrest, we designed studies with two pharmacological inhibitors of PI3K (Wortmannin and LY294002). Inhibition of PI3K/Akt pathway was unable to antagonize TRG-induced growth arrest or p21^CIP1 ^expression, suggesting these to be PI3K-independent effects of TRG. Wortmannin and LY294002 pretreatment however, antagonized TRG-induced down-regulation of p27^Kip1^, indicating PI3K involvement in regulating this. Since PI3K/Akt can down-regulate p27^Kip1 ^expression via phosphorylation and inhibition of FoxO transcription factors, (the known inducers of p27^Kip1 ^transcription) [36], and TRG treatment in serum-containing media results in increased FoxO1^Thr24^/FoxO3a^Thr32 ^phosphorylation (Figure 5B), it is conceivable that TRG utilizes this mechanism to decrease p27^Kip1 ^expression in HCC cells. In order to understand the mechanism by which TRG was inducing Akt^Ser473 ^phosphorylation, we focused on two kinases, mTORC2 and Pak, each one of which has been shown to function as PDK2 thus phosphorylating Akt at Ser473 position [38,39]. Although prolonged treatment with rapamycin was unable to antagonize TRG-induced Akt^Ser473 ^phosphorylation, these results don't completely rule out the participation of mTORC2 in mediating this, and more mechanistic approaches are needed to confirm this. Interestingly, these studies revealed the involvement of Pak in TRG-induced phosphorylation of Akt^Ser473^. Pak has been reported recently to be involved in PPARγ-induced motility of intestinal epithelial cells [44]. A recent study has demonstrated overexpression of Pak in HCC, which was also associated with a more aggressive behavior and cellular metastasis [45]. The involvement of Pak in breast cancer is also well established [46,47]. In addition, the knockdown studies with PPARγ-siRNA indicated the involvement of PPARγ in TRG-induced phosphorylation of Akt^Ser473^. Combined together, these suggested a potential crosstalk of PPARγ with Pak signaling in mediating Akt^Ser473 ^phosphorylation, which might explain the tumor promoting effects of PPARγ activation reported in earlier studies [15,16].

**Figure 9:**
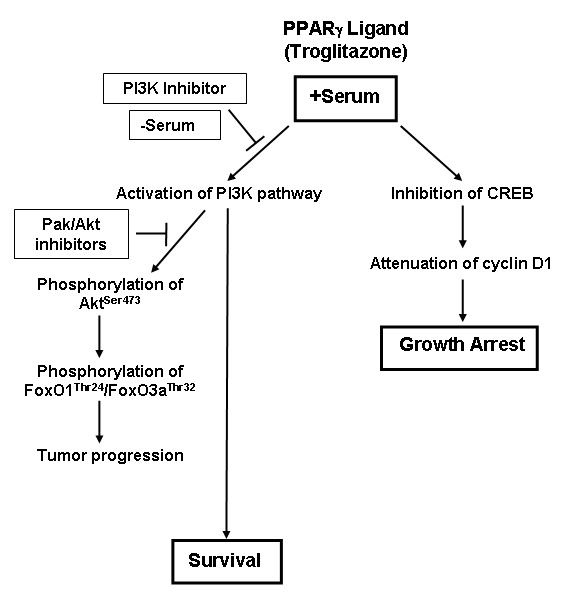
**Model representing the signaling pathway of TRG-induced cellular effects in HCC cells **. Incubation of the HCC cells with TRG in serum-containing media leads to a decrease in the expression of cyclin D1 resulting in cell growth arrest. TRG-induced reduction of cyclin D1 was shown to involve inhibition of CREB pathway in our earlier studies [32]. However, incubation with TRG under these conditions shows no apoptosis and leads to an increase in Akt^Ser473 ^and FoxO1^Thr24^/3a^Thr32 ^phosphorylation involving PI3K and Pak pathways, which might lead to tumor progression. Inhibition of PI3K pathway but not Pak or Akt pathways sensitizes cells towards apoptosis. In addition, incubation with TRG in serum-deficient media antagonizes Akt^Ser473 ^phosphorylation and leads to potent apoptosis.

Activation of PI3K/Akt axis is linked with inhibition of apoptosis and promotion of survival of cancer cells, suggesting that TRG treatment in these cells might lead to apoptotic resistance. In fact, TRG treatment under conditions that lead to growth arrest (i.e. in serum-containing media) was unable to induce any cleavage of PARP or Caspase-3 (mediators of apoptosis), suggesting absence of apoptosis. Surprisingly, the apoptotic potential of TRG was significantly increased when this ligand was added to the cells in a serum deficient media, associated with a large increase in PARP and Caspase-3 cleavage. In addition, TRG treatment under conditions that lead to apoptosis was associated with a dramatic decrease in Akt^Ser473 ^phosphorylation, suggesting an antagonism of PI3K/Akt axis. To determine whether activation of the PI3K/Akt signaling in the presence of serum might have antagonized the proapoptotic effects of TRG, studies were designed following pretreatment with the PI3K inhibitor LY294002. Pretreatment with LY294002 inhibited PI3K-mediated Akt^Ser473 ^and downstream FoxO1^Thr24^/FoxO3a^Thr32 ^phosphorylation and sensitized the cells towards TRG-induced apoptosis in the presence of serum. These studies provided evidence that TRG-induced apoptosis is modulated by PI3K pathway, an antagonism of which is required for induction of apoptosis. To understand the role of Akt in mediating this apoptotic response, TRG studies were also performed following antagonism of Akt pathway. Surprisingly, inhibition of Akt either by a pharmacological inhibitor or by siRNA-mediated knockdown of Akt1 and 2 expressions was unable to sensitize the cells towards TRG-induced apoptosis, when cultured in the presence of serum. Similarly, TRG was unable to induce apoptosis in MEFs derived from either Akt1-KO or Akt1&2-KO animals. These studies confirmed that activation of PI3K pathway can antagonize TRG-induced apoptosis in an Akt-independent manner. Elucidation of the mechanism by which serum deprivation converts TRG from a prosurvival to a proapoptotic molecule will be critical to understand the mechanism by which they regulate apoptosis and to utilize them in cancer therapy. Studies are currently underway to determine mechanistically whether the proapoptotic effects of TRG (in the absence of serum) involve PPARγ. Based on our studies, we have proposed a model describing the mechanism of TRG-induced cellular effects (Figure 9). The facts that (i) activation of PI3K/Akt axis is linked with many cancers and (ii) TRG treatment shows an activation of this axis, the long-term use of the Thiazolidinediones as type-II diabetic drugs raises an important clinical concern regarding their potential side effects in promoting cancer. Additional studies are also needed to understand whether the Thiazolidinediones currently used as type-II diabetic drugs (Rosiglitazone and Pioglitazone) produce similar effects as TRG on PI3K/Akt activation and apoptosis.

## Conclusions

The present study demonstrates that PPARγ ligand TRG when added in serum-containing media can inhibit cell proliferation in HCC cells independent of PI3K/Akt pathway. This is not associated with any apoptosis, while treatment with TRG in serum-deficient media results in potent apoptosis. Analysis of the signaling pathway(s) modulated under these two conditions revealed a TRG-mediated activation of PI3K/Akt signaling in serum-containing media which seems to involve the participation of Pak, and an inhibition of the same axis in serum-deficient media. In addition, pharmacological inhibition of PI3K sensitized the cells towards apoptosis in the presence of serum, suggesting involvement of PI3K signaling with this apoptotic resistance. However, inhibition of Akt by pharmacological inhibitor or knockdown by Akt-siRNA was unable to sensitize cells to TRG-induced apoptosis, suggesting this to be a novel PI3K-mediated Akt independent survival pathway. These studies suggest a potential mechanism by which PPARγ activation might lead to tumor promotion in certain cancer models, which might respond to a combination therapy with TRG and PI3K inhibitors. In addition, elucidation of the molecular mechanism that converts TRG to a proapoptotic molecule will help in increasing the efficacy of PPARγ ligands to be utilized in cancer therapy.

## Methods

### Reagents

The Huh-7 cells were obtained from Dr. Robert E Lanford (University of Texas Health Science Center, San Antonio) [48], the Hep3B cells were obtained from ATCC and mouse embryonic fibroblasts (MEFs) from Akt-wild-type (Akt-WT), Akt1-knockout (Akt1-KO), Akt1&2-Knockout (Akt1&2-KO) were obtained from Dr. Nissim Hay at University of Illinois, Chicago [49]. DMEM-F12, MEM, DMEM tissue culture media and LipofectAMINE 2000 were purchased from Invitrogen (Carlsbad, CA); Troglitazone, Wortmannin, LY294002, LY303511, Rapamycin, Akt inhibitor VIII and Pak inhibitor (PAK18) were purchased from Calbiochem, EMD Bioscience (San Diego, CA); the ELISA^PLUS ^kit was purchased from Roche Applied Sciences (Indianapolis, IN). The antibodies were obtained from the following sources: Poly (ADP-ribose) polymerase (PARP), Caspase-3, Akt, p21^Cip1^, pAkt^Ser473^, Akt1, Akt2, Akt3, cleaved Caspase-3, pFoxO1^Thr24^/3a^Thr32^, FoxO1, FoxO3a, pP70S6K^Thr389^, P70S6K, PPARγ from Cell Signaling Technology (Danvers, MA), Cyclin-D1 from Neomarkers, Lab Vision Corporation (Fremont, CA); GAPDH from Ambion Inc. (Austin, TX), p27^Kip1 ^from BD Biosciences (San Diego, CA), PCNA from Oncogene Research Products (Cambridge, MA).

### Cell culture

MEFs from Akt-WT, Akt1-KO, Akt1&2-KO, Huh-7 and Hep3B cells were grown in DMEM, DMEM-F12 and MEM medium respectively, supplemented with 10% FBS. All experiments were carried with subconfluent populations of cells. In the experiments with TRG treatment in serum-containing media, cells were treated with 25 μM TRG (unless indicated otherwise) in media containing 10% FBS for various lengths of time followed by either apoptosis assays or Western Blot analysis. In the studies with TRG treatment in serum deficient media, cells were treated with similar concentrations of TRG in media containing no serum.

### Cell Proliferation Assay

The cell proliferation assay was performed following protocols described earlier [32]. Briefly, subconfluent Huh-7 cells plated on 6-well plates were treated with either DMSO or 25 μM TRG for various lengths of time. At the time of harvest, the cells were trypsinized and counted using a hemocytomemeter. The cell numbers were represented as % control considering the DMSO treated sample of 24 hours as 100%. Cells were plated in triplicate for each time point and each experiment was repeated at least twice.

### Apoptosis Detection by Cell death Detection ELISA assay

This assay was performed utilizing the cell death detection ELISA^PLUS ^kit (Roche Applied Sciences, Indianapolis, IN) as per manufacturer's specification and as described previously [50,51]. Cells plated on 6-well plates were treated with indicated concentrations of TRG, following which both adherent and floating (apoptotic) populations were harvested. They were lysed in NP-40 lysis buffer and the nucleosomes in the supernatant were detected photometrically using an ELISA plate Reader (SpectraMax 190, Molecular Devices). The readings were expressed as degree of apoptosis considering the untreated control as 1.

### Western Blot analysis

Western Blot analysis was performed following treatment of cells with various agents and at different time intervals following procedures described earlier [50,52]. Equal amounts of total protein were fractionated by SDS-PAGE, transferred to PVDF membranes, followed by Western Blotting with the indicated antibodies. In the studies with kinase inhibitors, cells were pretreated with the respective inhibitors followed by treatment with TRG.

### Small interference RNA (siRNA) transfection

The following siRNA sequences were utilized in these studies; Akt-siRNA (sense 5'-UGCCCUUCUACAACCAGGAdTdT-3'), Akt-3m-siRNA (sense 5'-UGCCGUUCUUCAACGAGGAdTdT-3') [42], and PPARγ-siRNA (sense 5'-AACAGAUCCAGUGGUUGCAGAdTdT-3') [53]. The siRNA oligonucleotides along with the corresponding antisense oligonucleotide were synthesized from Dharmacon (Lafayette, CO). The control-siRNA was from Ambion (Austin, TX). siRNA transfection was performed using lipofectAMINE 2000 as per manufacturer's instructions, following procedures described previously [50]. Pilot experiments were performed first to optimize the amount and time of maximal protein knockdown. TRG treatment was performed following siRNA transfection during the period of maximal protein knockdown.

## List of Abbreviations

APC: Adenomatous Polyposis Coli; CDKI: Cyclin dependent kinase inhibitors; 15d-PGJ2: 15-deoxy-Δ12,14-prostaglandin J2; EMT: Epithelial to mesenchymal transition; GI: Gastrointestinal; HCC: Hepatocellular carcinoma; KO: knockout; MEFs: Mouse Embryonic Fibroblasts; mTORC1: mammalian target of rapamycin complex 1; mTORC2: mammalian target of rapamycin complex 2; Pak1: p21-activated kinase1; PARP: Poly (ADP-ribose) polymerase; PCNA: Proliferating cell nuclear antigen; PI3Kinase: Phosphatidylinositol-3 Kinase; PPARα: Peroxisome Proliferator-activated receptor alpha; PPARδ: Peroxisome Proliferator-activated receptor delta; PPARγ: Peroxisome Proliferator-activated receptor gamma; RXR: Retinoid X receptor; siRNA: Small interference RNA; TRG: Troglitazone; WT: Wild-type.

## Competing interests

The authors declare that they have no competing interests.

## Authors' contributions

PM has performed the studies related to the kinase inhibitors, siRNA transfection and MEFs. SKP performed the TRG time and dose course studies. RPT contributed to the writing, drafting and preparation of the manuscript and data analysis. AR helped with the analyses of the PI3K/Akt experiments and provided intellectual input in this collaborative study. BR contributed to the overall study design, interpretation of the results during all phases and drafted/edited the final manuscript, which was read and approved by the authors.
